# Oxidative Stress Induced by Lead and Antioxidant Potential of Certain Adaptogens in Poultry

**DOI:** 10.4103/0971-6580.72668

**Published:** 2010

**Authors:** M. Ratan Kumar, A. Gopala Reddy, Y. Anjaneyulu, G. Dilip Reddy

**Affiliations:** Department of Pharmacology and Toxicology, College of Veterinary Science, Rajendranagar, Hyderabad - 500030, India; Department of Veterinary Pathology, College of Veterinary Science, Rajendranagar, Hyderabad - 500030, India

**Keywords:** Amelioration by adaptogens, lead, oxidative stress

## Abstract

Effect of lead was studied for its action on antioxidant defense in broilers. A total of 225 one-day-old male broiler chicks (Vencobb strain) were divided randomly into 15 groups consisting of 15 chicks in each group. Group 1 was maintained on basal diet, group 2 on polyherbal formulation (PHF; stressroak), group 3 on shilajit, group 4 on amla, and group 5 on vitamin E + selenium (Se). Group 6 was maintained on lead for 42 days (6 weeks) and group 7 on lead for 28 days and subsequently on basal diet without lead for the remaining two weeks. Groups 8, 9, 10, and 11 were given lead along with PHF, shilajit, amla, and vitamin E + Se, respectively throughout the experiment for 6 weeks. Groups 12, 13, 14, and 15 were given lead containing diet for the first four weeks (28 days) and subsequently treated with PHF, shilajit, amla, and vitamin E + Se, respectively for the remaining two weeks. Antioxidant status of the birds was analyzed by assaying blood samples for glutathione (GSH) peroxidase, GSH reductase, and catalase at the end of fourth and sixth weeks, whereas Thiobarbituric acid reacting substances (TBARS) and GSH concentrations were estimated in liver homogenate at the end of the sixth week. The antioxidant defense parameters were significantly altered in toxic control groups indicating the possible oxidative damage caused by lead, whereas the parameters were normal in control groups 1 to 5 and other groups that were given the drugs in test, indicating their good ameliorating activity in oxidative stress.

## INTRODUCTION

The evidence of involvement of free radicals and reactive oxygen species (ROS) in the pathogenesis of number of diseases and toxicities is increasing constantly. ROS are involved in the biological damage induced by a number of therapeutic molecules, poisonous chemicals, and toxins.[1] Studies have reported that lead has a potential of inducing oxidative stress and acts as a catalyst in the oxidative reactions of biological molecules by producing free radicals ROS. Therefore, the toxicities to several organ systems associated with this metal might be due to oxidative tissue damage. To prevent peroxidative tissue damage, there are protective mechanisms *in vivo*, such as an enzymatic (antioxidant enzymes) and nonenzymatic (GSH) defenses. Lead interferes with the activities of different antioxidant defenses, and reduced amounts of antioxidants may contribute to damage to organ systems including liver, kidney, and nervous system.[2] Oxidative damage by free radicals can be prevented by the use of antioxidants.[3] Keeping the above facts in view, an experimental study was planned to evaluate the mechanisms of lead-induced oxidative stress and injury to the biological system, and to evaluate the prophylactic and therapeutic potential of polyherbal formulation (PHF; stressroak), shilajit, amla, and vitamin E + selenium (Se) against experimental lead toxicosis in broilers.

## MATERIALS AND METHODS

A total of 225 male broiler chicks (Cobb strain) of one-day-old were randomly divided into 15 groups of 15 chicks in each group. Feed and water were provided *ad libitum* throughout the experiment. Groups 1, 2, 3, 4, and 5 were maintained on basal diet control, PHF (stressroak; 100 ppm in feed), shilajit (100 ppm in feed), amla control (500 ppm in feed), and vitamin E + Se (300 + 0.3 ppm in feed), respectively, and groups 6 and 7 were the toxic controls that were kept on lead for 42 and 28 days, respectively. Groups 8, 9, 10, and 11 were given lead along with PHF, shilajit, amla, and vitamin E + Se, respectively for six weeks (1 – 42 days). Groups 12, 13, 14, and 15 were maintained on lead for the first four weeks and on PHF, shilajit, amla, and vitamin E + Se for the subsequent two weeks.

Birds of all the groups were vaccinated with New castle disease vaccine on 7^th^ and 28^th^ day and infectious bursal disease vaccine on 10^th^ day. Blood samples were drawn from the wing vein on 28^th^ and 42^nd^ day from the birds (identified by wing band numbers) in each group for assay of glutathione peroxidase (GSH-PX),[4] glutathione reductase (GSH-R),[5] and catalase.[6] The TBARS[7] and glutathione (GSH)[8] concentrations were estimated by using liver homogenate at the end of the sixth week. All the chemicals used in the study are of analytical grade and were procured from Qualigens Pvt. Ltd., Mumbai, India. The data were subjected to statistical analysis by applying ANOVA as per the standard methods of Snedecor and Cochran.[9] Differences between means were tested using Duncan’s multiple comparison test, and significance was set at *P*<0.05.

## RESULTS AND DISCUSSION

The results of the study are presented in Table 1. The activities of GSH-PX, GSH-R, and catalase and the concentration of TBARS were significantly (*P*<0.05) elevated, whereas the concentration of GSH was significantly (*P*<0.05) reduced in lead toxic control groups 6, 7, 12, 13, 14, and 15 at the end of fourth week. Groups 12, 13, 14, and 15 that were supplemented respectively with PHF (stressroak), shilajit, amla, and vitamin E + Se during the last two weeks following discontinuation of lead revealed a significant (*P*<0.05) improvement in these parameters at the end of sixth week as compared with groups 6 and 7.

**Table 1:** Results of oxidative stress

Group	GSH-PX activity (units/ml)		GSH-R activity (units/ml)		Catalase activity (moles/sec)		GSH (n moles/g protein	TBARS activity (nmoles/mg protein)
	4^th^ week	6^th^ week	4^th^ week	6^th^ week	4^th^ week	6^th^ week	6^th^ week	6^th^ week
Basal diet (1 – 42 d)	85.387	87.278	43.481	42.300	2.517	3.042	107.478	0.807
	±1.805^bA^	±1.446^bA^	±1.292^dA^	±0.998^dB^	±0.100^cdA^	±0.042^efB^	±0.57^h^	±0.03^c^
PHF (stressroak)(1 – 42 d)	68.080	70.799	21.237	19.170	1.906	2.129	122.028	2.010
	±1.681^aA^	±1.285^aB^	±0.682^aA^	±0.601^aB^	±0.020^aA^	±0.096^aB^	±0.72^j^	±0.05^h^
Shilajit(1 –42 d)	69.013	71.147	24.188	20.107	2.109	2.408	120.039	0.407
	±1.456^aA^	±1.474^aA^	±1.177^bA^	±0.777^abB^	±0.062^bA^	±0.065^bB^	±0.48^j^	±0.01^a^
Amla (1 –42 d)	70.045	72.134	27.271	22.196	2.401	2.609	115.927	0.502
	±1.440^aA^	±1.498^aA^	±0.922^cA^	±0.974^bB^	±0.098^cA^	±0.083^cB^	±0.68^i^	±0.00^ab^
Vitamin E+Se (1 –42 d)	71.990	74.165	28.999	25.023	2.608	2.807	116.058	0.604
	±1.495^aA^	±1.268^aA^	±0.587^cA^	±0.803^cB^	±0.114^dA^	±0.134^dB^	±0.74^i^	±0.01^b^
Lead (1 – 42 d)	130.523	139.503	76.801	86.065	3.396	3.830	68.325	1.202
	±9.643^fA^	±10.088^hB^	±2.225^hiA^	±2.497^lB^	±0.097^gA^	±0.049^kB^	±0.81^a^	±0.07^fg^
Lead (1 –28 d); basal diet (29 –42 d)	128.924	106.496	79.637	54.194	3.407	3.204	72.357	1.105
	±5.939^fA^	±3.379^dB^	±2.072^jA^	±1.528^hB^	±0.088^gA^	±0.032^fghB^	±1.22^b^	±0.07^ef^
Lead + PHF (stressroak) (1 –42 d)	108.228	112.383	60.127	64.097	2.800	3.339	95.953	0.913
	±1.060^cA^	±1.236^deB^	±1.260^eA^	±1.543^iB^	±0.127^eA^	±0.072^hiB^	±1.01^g^	±0.07^cd^
Lead +shilajit(1 –42 d)	111.223	115.294	63.204	67.258	3.112	3.508	92.089	1.066
	±1.265^cdA^	±1.404^efgB^	±1.257^fA^	±1.4089^jB^	±0.095^fA^	±0.085^jB^	±0.80^f^	±0.04^ef^
Lead +amla (1 –42 d)	115.078	119.232	65.109	69.094	3.315	3.701	87.989	1.201
	±1.662^deA^	±1.279^fgB^	±1.387f^fgA^	±0.663^jkB^	±0.087^gA^	±0.111^kB^	±1.11^de^	±0.04^fg^
Lead +vitamin E+Se (1 –42 d)	118.050	121.240	67.019	70.746	3.415	3.796	89.205	1.318
	±0.920^eA^	±1.299^gB^	±1.450^gA^	±1.616^kB^	±0.070^gA^	±0.091^kB^	±0.95^e^	±0.10^g^
Lead – 28 d); PHF (stressroak) (29 – 42 d)	129.326	89.007	78.308	44.114	3.301	2.912	85.290	0.806
	±4.765^fA^	±1.969^bcB^	±1.945^ijA^	±1.222^deB^	±0.065^0.065^	±0.142^deB^	±1.37^c^	±0.03^c^
Lead – 29 d);shilajit (29 – 42 d)	111.081	114.955	75.046	45.135	3.401	3.074	88.326	0.907
	±1.781^cdA^	±1.476^efB^	±0.905^hA^	±1.150^efB^	±0.076^gA^	±0.138^efgB^	±0.80^de^	±0.04^cd^
Lead (1 – 28 d);amla (29 – 42 d)	129.322	93.330	77.397	47.203	3.409	3.208	85.927	1.018
	±3.335^fA^	±1.175^bcB^	±0.935^hijA^	±1.300^fgB^	±0.108^gA^	±0.070^ghB^	±0.51^cd^	±0.06^de^
Lead (1 –28 d); vitamin E + Se (29 – 42 d)	130.597	94.911	78.331	49.211	3.349	3.419	84.992	1.111
	±3.625^fA^	±1.391^cB^	±1.357^ijA^	±1.863^gB^	±0.088^gA^	±0.059^aB^	±1.05^c^	±0.07^ef^

Values are mean + SE of six observations; Means with different alphabets as superscripts differ significantly (*P*<0.05) ANOVA; Capital alphabets (horizontal comparison); small alphabets (vertical comparison);GSH-PX - glutathione peroxidase; GSH-R - glutathione reductase; TBARS - Thiobarbituric acid reacting substances; PHF - polyherbal formulation; Se – selenium; SE - Standard error

The concentration of GSH in liver was assessed, as it is the major organ involved in xenobiotic metabolism. TBARS in liver tissue were analyzed to determine the extent of peroxidative stress, and the activities of GSH-PX, GSH-R, and catalase were assessed, as they form the major components of antioxidant defense system in the living. The activities of GSH-PX, GSH-R, and catalase in blood and TBARS activity in liver were significantly increased in toxic groups, whereas the concentration of GSH was reduced suggesting the ongoing peroxidative stress. Recent studies have shown that lead causes oxidative stress by inducing the generation of ROS, reducing the antioxidant defense system of cells by depleting GSH, inhibiting sulfhydryl-dependent enzymes, interfering with certain essential metals needed for antioxidant enzyme activities, and/or increasing susceptibility of cells to oxidative attack by altering the membrane integrity and fatty acid composition. Consequently, it is possible that impaired oxidant/antioxidant balance could be partially responsible for the toxic effects of lead. Enhanced oxidative stress contributes to lead-induced toxicity, where restoration of a cell’s antioxidant capacity appears to provide a partial remedy. Several studies are underway to determine the effect of antioxidant supplementation following lead exposure. Data suggest that antioxidants may play an important role in abating some hazards of lead.[10] All the changes in the antioxidant defense profile were significantly reversed when treated with PHF (stressroak), shilajit, amla, and vitamin E + Se. The beneficial effects of PHF (stressroak) are attributed to antioxidant and antistress principles namely, *Withania somnifera*,[11] *Ocimum sanctum*,[12,13] *Phyllanthus emblica*,[14] *Mangifera indica*,[15] and shilajit.[16] The withanolides present in *Withania somnifera* are known to inhibit lipid peroxidation by their antioxidant properties that break the chain reaction.[17] The *Ocimum sanctum* has been reported to reduce lipid peroxidation and increase the GSH concentration in blood.[18] Gallic acid and geraniin, which are the active principles of *Phyllanthus emblica*, have been reported to possess strong nitric oxide (free radical) scavenging activity.[19] In a study, Hernandez *et al*.[20] reported that *M. indica* extract attenuated accumulation of ROS. Se is considered as an essential component of GSH-PX, which is the major intracellular antioxidative enzyme that catalyses the reduction of hydrogen peroxide and organic hydroperoxides to nontoxic compounds. Vitamin E, which is abundant in several natural sources, has free radical quenching activity.[13] Several reports have demonstrated an antioxidant synergism between vitamin E and Se in counteracting free radical-induced oxidative stress in the biological system.[21]

From this study, it can be concluded that lead-induced damage to the biological system is attributed to the excess generation of free radicals and impairment of antioxidant defenses, and supplementation of PHF (stressroak), shilajit, amla, and vitamin E + Se either prophylactically or therapeutically could significantly reverse the toxic effects of lead.
